# Tuning biodegradability, bone-bonding capacity, and wear resistance of zinc-30% magnesium intermetallic alloy for use in load-bearing bone applications

**DOI:** 10.1038/s41598-024-52648-6

**Published:** 2024-01-29

**Authors:** Rasha A. Youness, Mohammed A. Taha

**Affiliations:** 1 Spectroscopy Department, National Research Centre, El Buhouth St., Dokki, Giza, 12622 Egypt; 2 Solid State Physics Department, National Research Centre, El Buhouth St., Dokki, Giza, 12622 Egypt

**Keywords:** Biophysics, Materials science

## Abstract

This work aimed to improve the rapid biodegradation, poor wear resistance properties, and lack of bioactivity of metallic biomaterials to be used in orthopedic applications. In this context, zinc–magnesium (Zn–Mg) alloy with successive contents of calcium silicate (CaSiO_3_) and silicon nitride (Si_3_N_4_) was prepared using powder metallurgy technique. After sintering, their phase composition and microstructure were investigated using the X-ray diffraction technique and scanning electron microscopy (SEM), respectively. Furthermore, their degradation behavior and ability to form hydroxyapatite (HA) layer on the sample surface after immersion in simulated body fluid (SBF) were monitored using weight loss measurements, inductively coupled plasma-atomic emission spectroscopy, and SEM. Moreover, their tribo-mechanical properties were measured. The results obtained showed that the successive contents of CaSiO_3_ were responsible for improving the bioactivity behavior as indicated by a good formation of the HA layer on the samples’ surface. Additionally, ceramic materials were responsible for a continuous decrease in the released ions in the SBF solution as indicated by the ICP results. The tribology properties were significantly improved even after exposure to different loads. Based on the above results, the prepared nanocomposites are promising for use in orthopedic applications.

## Introduction

The need for bone tissue to be repaired, organ functions to be restored, and aesthetic restoration is rising as a result of population aging and a rise in life expectancy^1–4^. In this regard, since 1895, when metallic biomaterials were first developed, they have continued to advance^5^. These materials are widely used in bone replacement applications because of their adequate strength, which gives us the chance to employ them as load-bearing implants in orthopedics and dentistry. The main drawbacks of these materials, however, include their non-biodegradability in the biological environment^6–8^, allergenicity, toxicity of their released ions, poor wear resistance, poor bending ductility, and lack of bioactivity which is necessary to form a firm chemical bond with surrounding tissues^9,10^.

Due to its combination of biocompatibility, biodegradability, and greater mechanical similarity to those of cortical bone than to any other metallic or polymer implant materials, Mg alloys have been researched recently for biodegradable bone implant applications^11,12^. However, the comparatively weak corrosion resistance of pure Mg makes it difficult to utilize; as a result, the mechanical characteristics of the implant dramatically deteriorate and the tissues are unable to mend. Additionally, Mg corrosion produces hydrogen (H_2_) gas, which accumulates near the implant in vivo. Another concern with these uses is that Mg alkalizes surrounding tissues due to H_2_ gas^13,14^. To satisfy the needs of both implant biodegradation and new bone growth, it is crucial to strengthen the corrosion and mechanical resistance of Mg and its alloys^15^.

Biodegradable Zn alloys have advanced significantly in recent years due to their biological significance in bone metabolism, which includes their capacity to promote the production of osteoblasts while simultaneously inhibiting the differentiation of osteoclasts and enhancing bone strength. Therefore, it would seem that biodegradable Zn alloys offer distinct advantages over biodegradable polymers and Mg alloys in orthopedic applications^16–18^. In this regard, new alloy systems, such as Zn–Mg^19,20^ and Zn–Li^21^ alloys, have been developed; nevertheless, not many opaque objects have yet been produced with them. This lack of interest in Zn alloys by researchers may be brought on by their low hardness and lack of bioactivity behavior.

As a result of its outstanding properties including bioactivity and biocompatibility, calcium silicate bioceramics are now thought of as a new and advanced class for usage in orthopedic and dental applications. The main issues limiting their use in clinical applications are their high degradation rate, which can raise the pH value of the physiological environment and adversely affect their capacity for osseointegration, inability to support osteoblast proliferation, and low mechanical properties^22–24^.

A great material for applications requiring high strength at high temperatures, abrasion resistance, good oxidation resistance, and reasonably high toughness is Si_3_N_4_ due to its special mix of characteristics^25–27^. In this regard, Si_3_N_4_ is suitable for use as an orthopedic biomaterial due to the above-mentioned advantages along with its good biocompatibility^28,29^. Based on the above facts, the combination of these materials may be beneficial in the application of bone replacement to take advantage of their advantages and reduce their disadvantages, thanks to the mechanical alloying (MA) technique that will be effective in obtaining good homogeneity and optimal interaction between these materials^30–32^.

Many researchers have prepared Zn–Mg or Mg–Zn alloys for orthopedic and vascular procedures using conventional casting^33–35^. To the authors’ knowledge, these experiments have not been expanded to include the addition of other materials, such as ceramics, to overcome the fundamental obstacles that prevent their use in clinical research. Hence, this study presents a novel solution to these defects by adding durable ceramic, i.e., Si_3_N_4_, and a bioactive ceramic, i.e., CaSiO_3_, and alloying them with Zn–Mg alloy to improve the tribo-mechanical, biodegradation, and bioactivity properties of these composites. (24 Jan 2024).

## Materials and methods

### Materials

Zn (99.5%), Mg (99.6%), CaSiO_3_ (99.2%), Si_3_N_4_ (98.5%), sodium chloride (NaCl; 99%), sodium bicarbonate (NaHCO_3_; 99.3%), potassium chloride (KCl; 99.2%), calcium chloride (CaCl_2_; 99.4%), dibasic potassium phosphate (K_2_HPO_4_; 99.4%), magnesium chloride (MgCl_2_; 99%), tris-hydroxymethyl-amino-methane ((CH_2_OH)_3_CNH_3_), and hydrochloric acid (HCl; 99.2%) have been supplied from Sigma–Aldrich. High-resolution transmission electron microscopy (HRTEM; JEOL–JEM2100, Japan, operated at an accelerating voltage of 120 kV) was employed to investigate the particle size of the reinforcements used in the preparation of samples, i.e., CaSiO_3_ and Si_3_N_4_.

### Methods

#### Preparation of Zn–Mg alloy and its ceramics-containing nanocomposites powders

For the preparation of Zn–Mg alloy, referred to in the text as ZM1, both Zn and Mg were weighed at a ratio of 70:30 vol% and mixed thoroughly using a high-energy ball mill (HEBM) for 15 h with a ball-to-powder ratio (BPR) of 1:3 and 120 rpm as a rotation speed. Then, milling was carried out for 20 h with a BPR of 1:15 and a milling speed of 350 rpm. In the case of preparing other ceramics-containing Zn–Mg alloys, denoted as ZM2, ZM3, ZM4, and ZM5, the used parameters are also applied. Notably, HRTEM was used to examine the produced nanocomposites. It should be noted that Table 1 displays the makeup of the samples that were generated and their acronyms.

**Table 1:** Scheme of the prepared nanocomposites referring to the sample code and its composition (vol%).

Sample code	Zn–Mg alloy	CaSiO_3_	Si_3_N_4_
ZM1	100	0	0
ZM2	95	5	0
ZM3	94	5	1
ZM4	88	10	2
ZM5	76	20	4

#### Sintering process

**Figure 1:**
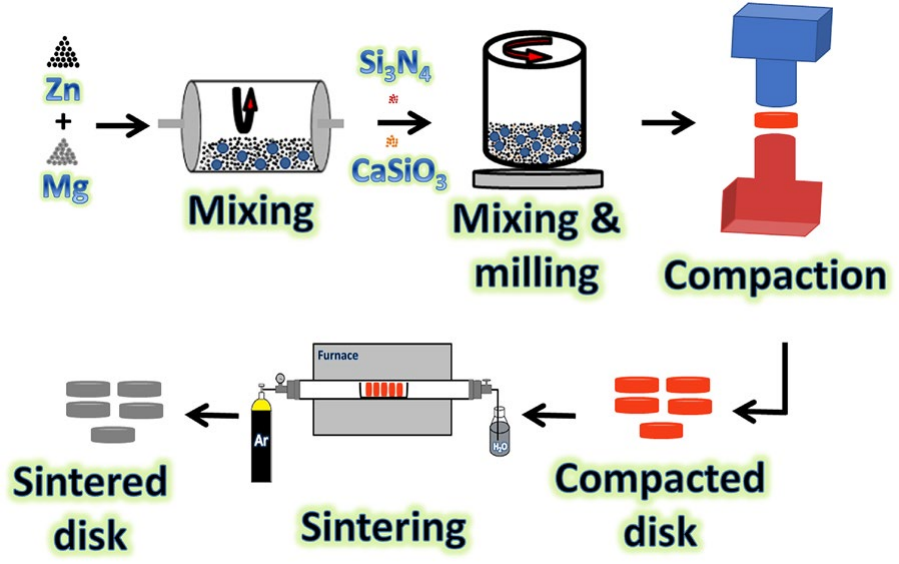
The schematic diagram represents the steps for preparation of Zn–Mg-based alloy and its composites.

**Figure 2:**
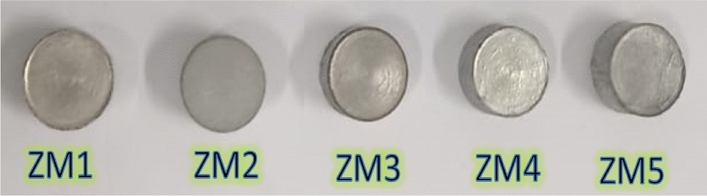
Photographs showing the prepared samples.

Using a hydraulic press at 30 MPa and a heating rate of 5 °C/min for 1 h at 360 °C, the ground powders were compressed into pellets measuring 16 mm in diameter and 4 mm in length. It is important to observe that Figs. 1 and 2, respectively, exhibit the sample preparation procedures together with the images illustrating them.

#### Characterization of the prepared alloy and its composites

Using the XRD technique, the phase composition of each produced sample was investigated. FESEM coupled with energy dispersive X-ray analysis **(**EDX) (type Quanta FEG250 with an accelerating voltage of 30 kV and a magnification of 10× up to × 300,000) was also used to examine their microstructure.

#### Evaluation of the biological properties of the prepared alloy and its composites

##### Bone bonding capacity

By soaking the obtained specimens for 14 days in a SBF made by the instructions provided by Kokubo et al.^36,37^, while preserving the ratio of sample grains to the volume of solution = 0.01 cm^3^^38^, the in vitro bioactivity of the specimens was evaluated. The modifications that took place on the samples’ surfaces were then examined using FESEM on the soaked samples.

##### Biodegradability tests

Weight loss measurements were used to determine how the samples behaved during biodegradation in SBF solution. The following equation was used to compute the percentage of weight loss following an immersion period (t):

1 $$ Weight\;loss\left( \% \right) = \frac{{{\text{Wi}} \times {\text{Wt}}}}{Wi} \times Wi $$

where W_i_ is the initial weight of each sample, as a disc, (i.e. before soaking) and W_t_ is the weight of the sample disc after soaking in the SBF for a period of time (t) after it has been dried. Weight loss measurements were performed in triplicate.

The concentrations of Zn, Mg, Si, calcium (Ca), and phosphorus (P) ions in the SBF solution were measured before and after the samples were soaked in the solution for 14 days using the ICP-AES (Jobian Yvon Horiba Ultima 2000).

##### Evaluation of cytotoxic activity of samples prepared using a human osteosarcoma cell line

The human osteosarcoma Saos-2 cell line (ATCC, USA) was used by the Bioassay–Cell Culture Laboratory, National Research Centre, Egypt to assess the cytotoxic activity of the specimens under study. With a reference wavelength of 620 nm, the absorbance was determined using a microplate multi-well reader (Bio-Rad Laboratories Inc., model 3350, Hercules, California, USA). On the other hand, the statistical significance between the specimens and the negative control was examined, and probit analysis for IC50 and IC90 was carried out using the SPSS11 software. However, the following formula was used to calculate the viability change percentage:

2 $$ \left( {{\text{Reading}}\;{\text{of}}\;{\text{extract}}/{\text{Reading}}\;{\text{of}}\;{\text{negative}}\;{\text{control}}} \right) - {1}) \times {1}00 $$

#### Measurement of the different properties of the obtained samples

##### Physical properties

As mentioned in Refs.^39,40^, the Archimedes technique (ASTM: B962-13) was used to examine the bulk density, apparent porosity, and relative density of all sintered solids.

##### Mechanical properties

The microhardness of the prepared specimens was assessed by ASTM: B933-09, as stated in Refs.^32,41^. It should be emphasized that for each data point, at least five indentations per specimen were measured. Furthermore, the ultrasonic wave velocities (longitudinal and shear) propagated in the samples were measured at room temperature using a pulse-echo approach MATEC Model MBS8000 DSP (ultrasonic digital signal processing) system with a 5 MHz resonating frequency. To summarize, both longitudinal (V_L_^2^) and shear (V_S_^2^) ultrasonic velocities may be used to calculate the values of Lame’s constants, i.e., λ and μ as follows:

3 $$ {\uplambda } = {\uprho }\left( {V_{L}^{2} - 2{\text{V}}_{S}^{2} } \right) $$

4 $$ {\upmu } = {\uprho }V_{S}^{2} $$

5 $$ G =\upmu $$

6 $$ E =\upmu \frac{{3\lambda + 2\upmu }}{{\lambda +\upmu }} $$

7 $$ B = \lambda + \frac{2}{3}\upmu $$

where *ρ* is the measured density of samples, G is the shear modulus, E is Young’s modulus, and B is the bulk modulus.

##### Tribology tests

The nanocomposite samples underwent a wear test under dry sliding conditions at room temperature using a pin-on-disk wear-testing system. The wear test used process settings including a speed of 0.8 m/s, a sliding distance of 250 m, and various applied loads of 10, 20, and 40 N. The wear rate (W) of sintered nanocomposite samples was determined using the following equation^42^:

8 $$ {\text{W}}\left( {\frac{{mm^{3} }}{{{\text{Km}}}}} \right) = \frac{{\text{M}}}{{{\uprho } \times {\text{D}}}} $$

where M is the weight loss (g), ρ is the bulk density (g/mm^3^) and D (m) is the sliding distance (m).

It is important to note that the wear tracks for the ZM1 and ZM5 samples, as representative of the whole group of samples, were examined using FESEM.

##### Statistical analyses

Statistical analyses of the results of the physical, mechanical, and tribological properties were performed with the help of Origin Lab Pro to determine the mean value, standard deviation, and variance of the five readings taken for each test.

## Results and discussion

### Characterization of nanopowders and their particle size using HRTEM

**Figure 3:**
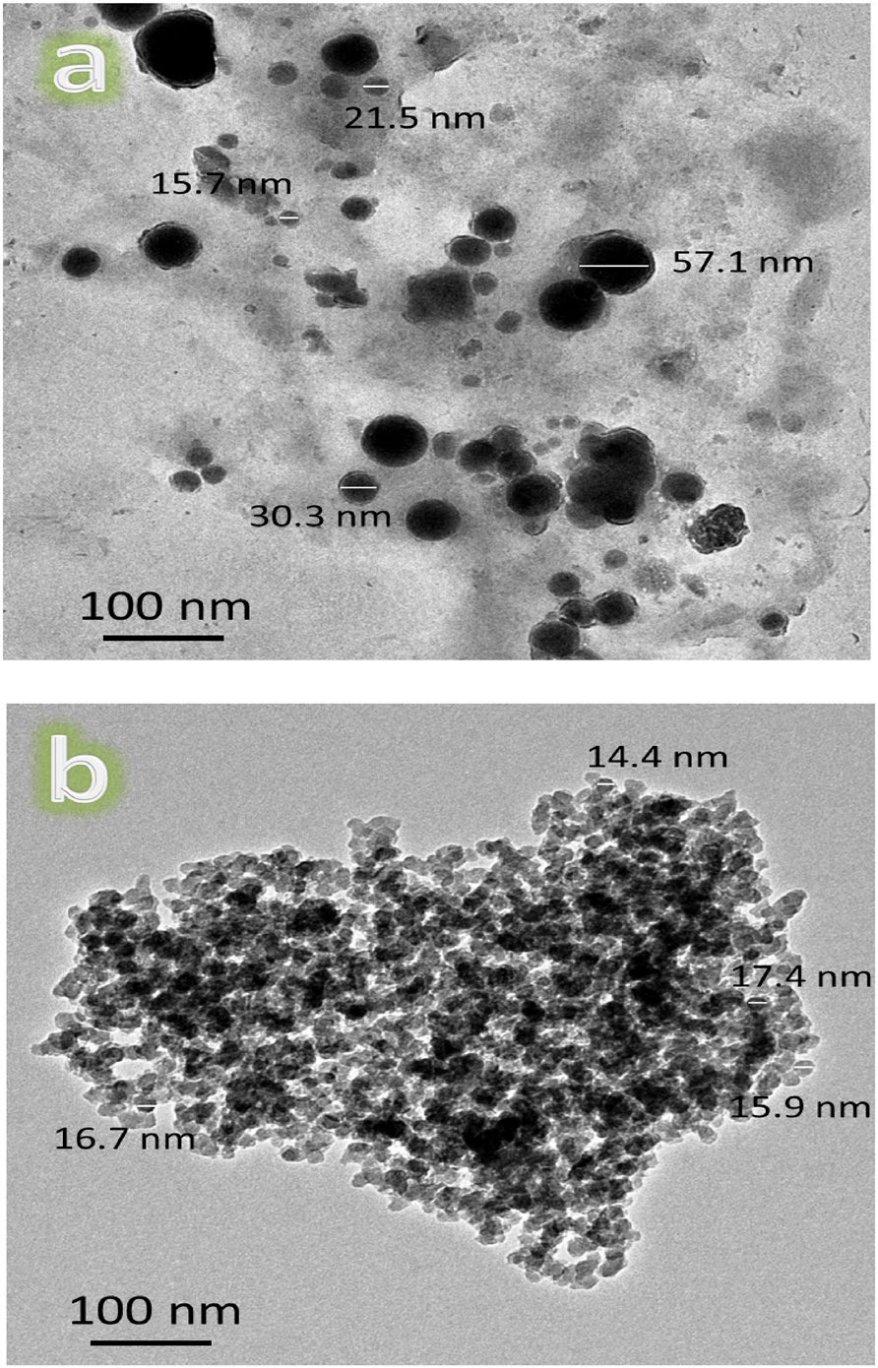
HRTEM images of reinforcements, i.e., (**a**) CS and (**b**) Si_3_N_4_ used in the preparation of Zn–Mg-based alloy composites.

**Figure 4:**
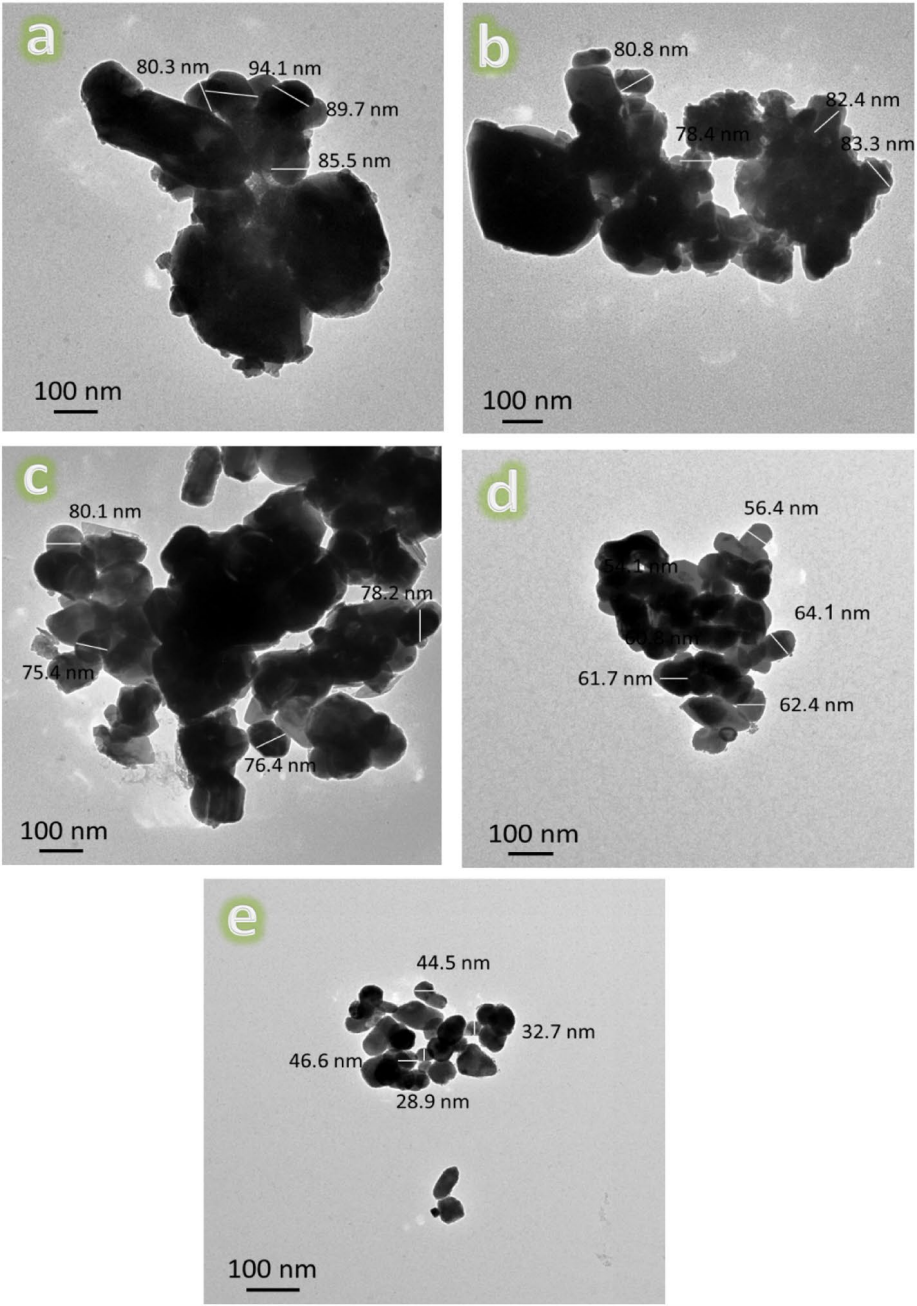
HRTEM images of the fabricated (**a**) ZM1, (**b**) ZM2, (**c**) ZM3, (**d**) ZM4, and (**e**) ZM5 samples.

**Figure 5:**
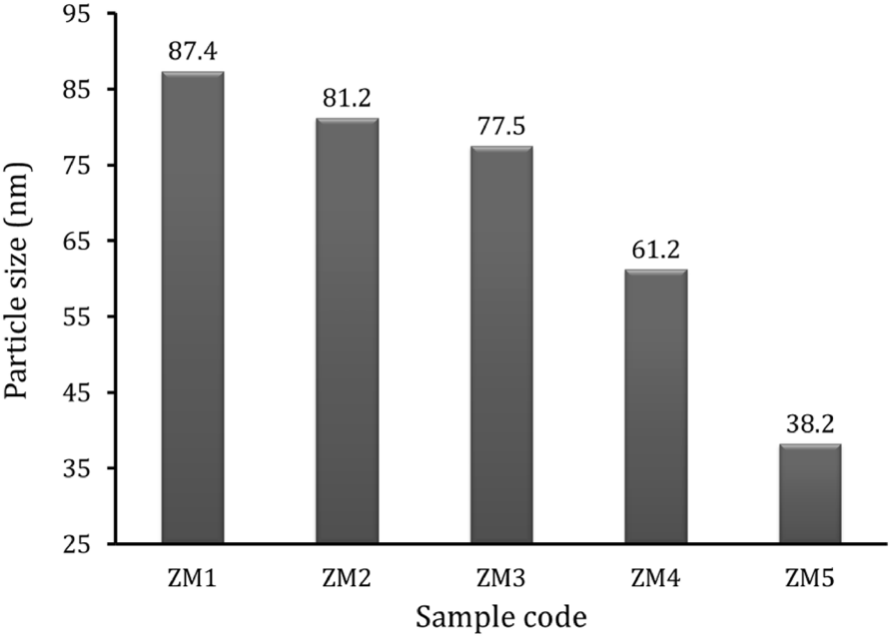
Average particle sizes of all prepared samples measured from HRTEM.

After 20 h of milling, the HRTEM images of the hybrid reinforcements (CaSiO_3_ and Si_3_N_4_) employed and the powder of nanocomposites, namely ZM1, ZM2, ZM3, ZM4, and ZM5, are shown in Figs. 3a, b and 4a–e. Figure 5 also depicts the nanocomposite powder particle size as determined by HRTEM image analysis. Figure 3 makes it clear that the CaSiO_3_ and Si_3_N_4_ particles are spherical and measure around 31.15 and 16.1 nm, respectively.

It can be seen from Fig. 4a that the Zn–Mg alloy, ZM1 sample, shows the highest agglomeration among the investigated samples. While successive increases in the volume percentages of the reinforcements, i.e., CaSiO_3_, Si_3_N_4_, as observed from Fig. 4b–e, led to a gradual decrease in this agglomeration. This remarkable improvement in agglomeration was not the only improvement that occurred in the prepared nanocomposites as a result of the successive addition of ceramics, as it also significantly reduced their particle sizes, as shown in Fig. 5. The detected average particle sizes of the ZM1, ZM2, ZM3, ZM4, and ZM5 samples are 87.4, 81.2, 77.5, 61.2, and 38.2 nm, respectively.

It is because the Zn–Mg alloy particles (which are ductile) deform while the CaSiO_3_ and Si_3_N_4_ (which are hard) break apart. This is why the particle sizes of the nanocomposites that are made by successively embedding ceramic components are getting smaller. Therefore, when the balls collide, the Zn–Mg alloy particles begin to weld, and the ceramic particles interfere with two or more matrix particles. So, the ceramic particles stay near the edges of the Zn–Mg alloy particles that have been joined together. This makes nanocomposites powders with particles that are much smaller. In addition, this successive addition of ceramic materials to the Zn–Mg alloy reduces the resulting agglomeration. Based on this fact, the reader can expect the importance of adding ceramic materials during alloy making. These findings are quite consistent with those in Refs.^43–45^.

### Characterization of the produced alloy and its composite materials

#### Composition of phases

**Figure 6:**
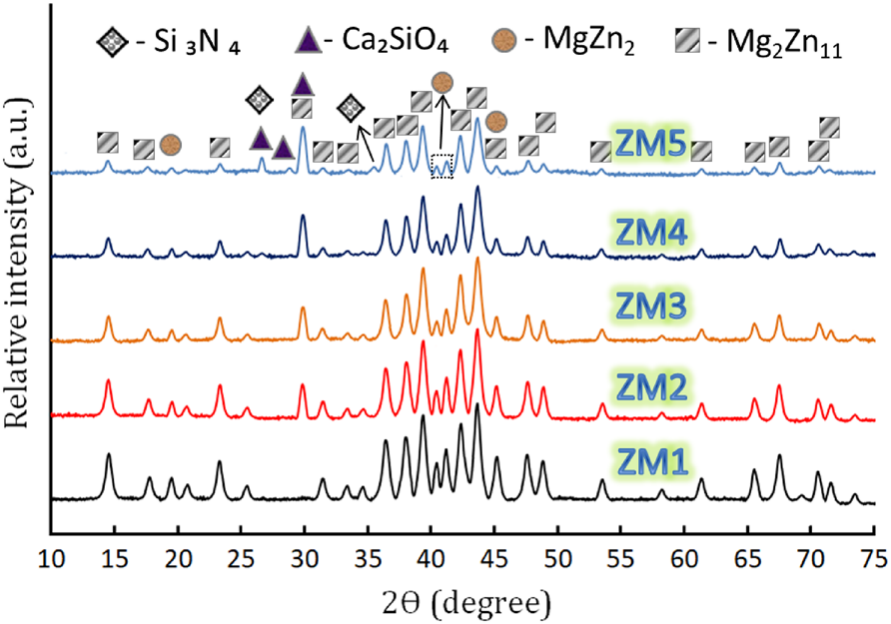
XRD patterns of all samples after being heat–treated at 360 °C for 1 h.

To analyze the generated Zn–Mg alloy and its composites resulting from the sintering process at 360 °C for 1 h as shown in Fig. 6, XRD technique was employed. From Fig. 6, it is possible to see that the ZM1 sample consists of MgZn_2_ and Mg_2_Zn_11_ phases, which are identified according to the ICCD file cards: 72–1177 and 06–0664, respectively. This means that the as-prepared Zn–Mg alloy is an intermetallic alloy which gives it valuable advantages over the conventional alloy. The difference between these types can be understood with the help of Ref.^46^ as follows:

Two main categories of materials may be created from two or more metallic elements: alloys and intermetallics. The elemental crystal structure of one of the component elements is adopted in alloys, also known as solid solutions. Alloys are metal mixes that are at random. The compounds known as intermetallics have a known stoichiometry, crystal structure, and particular locations designated for the atoms of each component element. Intermetallics include the controlled, rather than random, positioning of two or more different atom kinds. Because of this fact, the intermetallic alloy can be distinguished from a conventional alloy by such ordering. Due to their high internal order and mixed (metallic and covalent/ionic) bonding, which are absent in alloys, intermetallics exhibit unique magnetic, superconducting, mechanical, and chemical characteristics. Another advantage of intermetallic alloys is that they possess higher thermal stability than conventional alloys, which prevents phase separation. Last but not least, an intermetallic alloy would guarantee homogeneity thanks to its regular structure.

In the XRD pattern of the ZM2 sample, the new phase; namely CaSiO_3_ begins to appear, which is identified according to the ICCD file card: 84–0655, and the intensities of the other phases decrease slightly. Because the content of Si_3_N_4_ added to the as-prepared nanocomposites was only 1 vol%, which is below the XRD instrument’s detection limit, the situation almost did not alter in the ZM3 sample. Surprisingly, the Si_3_N_4_ phase is undetectable in the XRD device, while the CaSiO_3_ phase becomes increasingly apparent in the ZM4 sample as its quantity increases from 5 to 10 vol%. Under the effect of increasing Si_3_N_4_ content to 4 vol%, this phase begins to appear in the ZM5 sample, as can be identified by the ICCD file card: 83–0701. In addition, a better appearance of the CaSiO_3_ phase and an appreciable decrease in the intensity of the MgZn_2_ and Mg_2_Zn_11_ phases was seen. A crucial indicator of the absence of contamination that could occur during the grinding and/or sintering processes is the absence of other phases.

Table 2 lists the impact of ceramics on the Zn–Mg alloy’s crystal size, lattice strain, and dislocation density. This table shows that as the concentrations of CaSiO_3_ and Si_3_N_4_ rise, the crystallite size decreases while the lattice strain and the dislocation density rise. ZM1, ZM2, ZM3, ZM4, and ZM5 specimens have computed crystal sizes of 27.81, 25.46, 23.78, 20.11, and 15.54 nm, respectively, whereas the dislocation density of the same samples is 1.29 × 10^−3^, 1.54 × 10^−3^, 1.77 × 10^−3^, 2.47 × 10^−3^, and 4.14 × 10^−3^%, respectively.

**Table 2:** Crystal size, lattice strain, and dislocation density for all samples prepared.

Sample	Crystal size (nm)	Lattice strain (%)	Dislocation density (%)
ZM1	27.81	0.3762	1.29 × 10^−3^
ZM2	25.46	0.4110	1.54 × 10^−3^
ZM3	23.78	0.4399	1.77 × 10^−3^
ZM4	20.11	0.5202	2.47 × 10^−3^
ZM5	15.54	0.6733	4.14 × 10^−3^

#### Surface morphology

**Figure 7:**
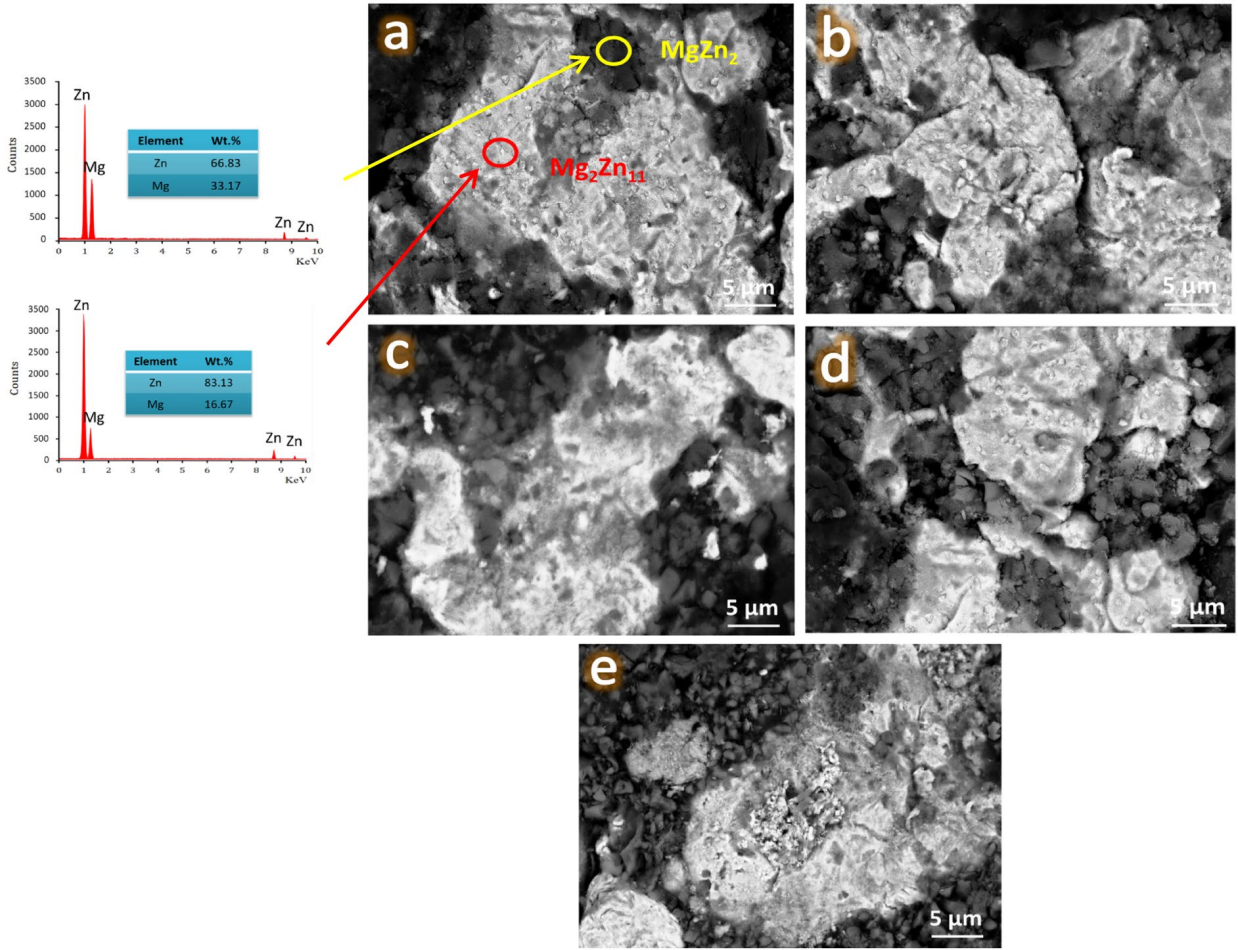
FESEM images of (**a**) ZM1 along with the EDX spectra of the resultant MgZn_2_ and Mg_2_Zn_11_ phases, (**b**) ZM2, (**c**) ZM3, (**d**) ZM4, and (**e**) ZM5 samples after being heat–treated at 360 °C for 1 h.

**Figure 8:**
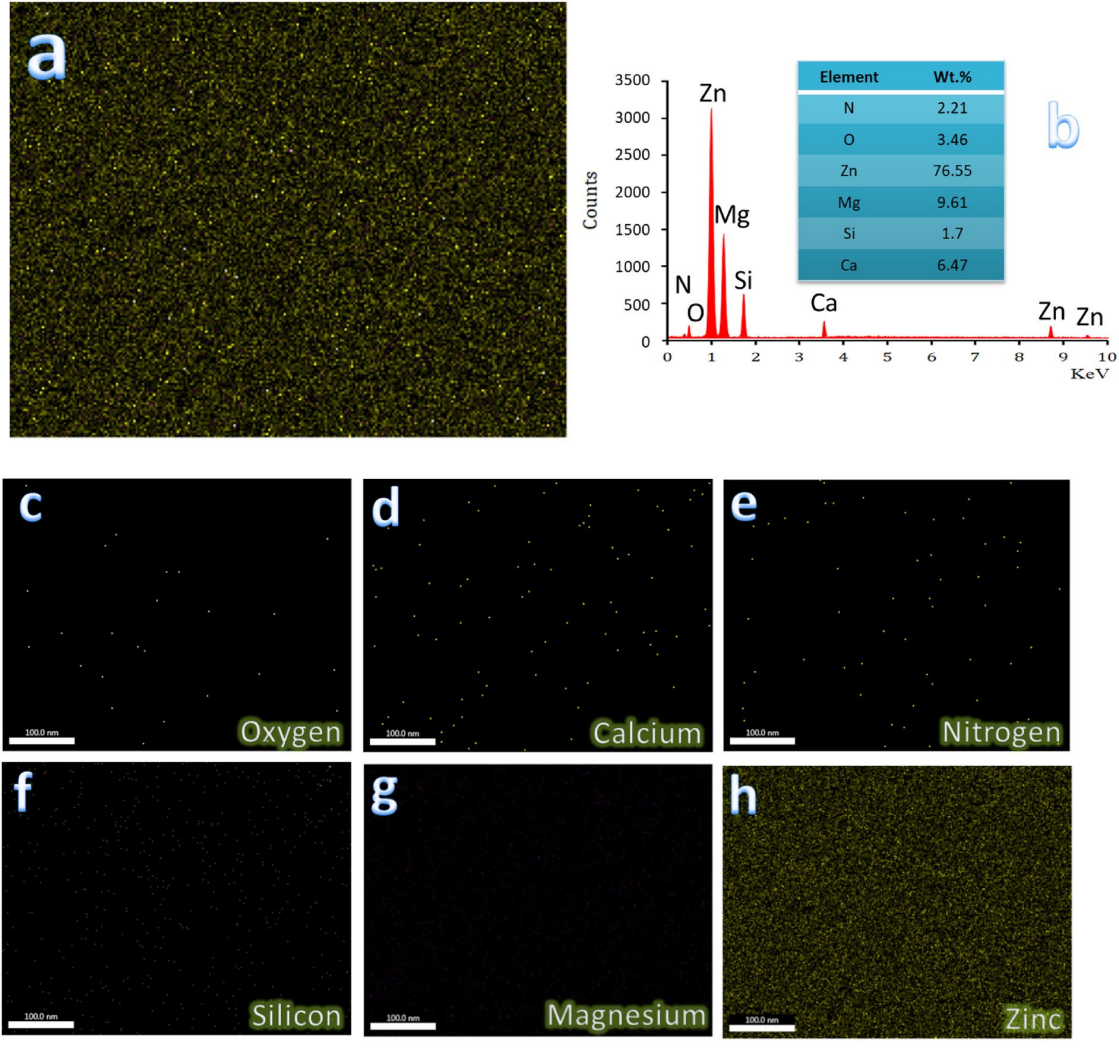
(**a**) EDX mapping of all constituents of ZM5 sample, (**b**) EDX spectra of ZM5 sample and elemental mapping of the constituents forming ZM5 sample, i.e., (**c**) zinc, (**d**) magnesium, (**e**) calcium, (**f**) silicon, and (**g**) nitrogen.

The microstructure of all samples sintered at 360 °C for 1 h along with the EDX pattern of the ZM1 sample was determined using FESEM-EDX as illustrated in Fig. 7a–e. Furthermore, the EDX mapping of all constituents in the ZM5 sample, the EDX spectra of the ZM5 sample, and the elemental mapping of the constituents forming the ZM5 sample, i.e., Zn, Mg, Ca, Si, and N are represented in Fig. 8a–h.

Due to the small particle size of both the as-prepared nanocomposite powders and the reinforced ceramics, a high magnification power was used in this study to highlight the occurrence of condensation in the microstructure of the sintered samples. This is in line with the outcomes depicted in Figs. 3, 4 and 5. It is evident that the selected sintering temperature, which is about 86% of the melting point of Zn, the alloy’s matrix, achieved excellent condensation behavior for the sintered ZM1 sample. It is crucial to emphasize that Fig. 7a also demonstrates the existence of two phases in the microstructure, supporting the findings from the XRD patterns covered in the preceding section. This conclusion is highly supported by the EDS pattern of the ZM1 sample that confirms the formation of the mentioned phases. Interestingly, the addition of ceramics leads to a steady decrease in the condensation behavior of the investigated samples. To provide the reader with more details, in sample ZM2, a few CaSiO_3_ particles develop with a slight decrease in sample condensation. This reduction in condensation persists as the amount of ceramics in the composites rises. As anticipated, ZM3 exhibits no discernible variation from ZM2, which is in excellent accordance with the findings described in the XRD section. This fact is supported by the discovery that there is less diffusion between the ceramic particles, allowing them to be exhibited independently since the sintering process is carried out at a lower temperature than that needed for ceramics. Notably, CaSiO_3_ and Si_3_N_4_ have melting points of 1540 and 2830 °C, respectively. Noticing this big difference between the melting points of ceramics and the prepared intermetallic alloy, the reader expects a much lower densification than what appears in the images, but this did not happen, thanks to the fact that the particle size of the added ceramics is in the nanometer range, which facilitated the diffusion process and obtaining a better condensation than expected. Due to the sample’s double concentration of CaSiO_3_ and Si_3_N_4_, this matter is more obvious in the ZM5 sample.

Based on the data obtained from Fig. 8a–h, one can conclude that there are no other components, and therefore the presence of contamination during the grinding or sintering operations can be ruled out, which supports the findings presented in “Composition of phases” section. Another conclusion obtained from this figure is that a well-homogeneous distribution of ceramic materials in the Zn–Mg alloy is obtained.

### Evaluation of the biological properties of the prepared alloy and its composites

#### Bone bonding capacity

**Figure 9:**
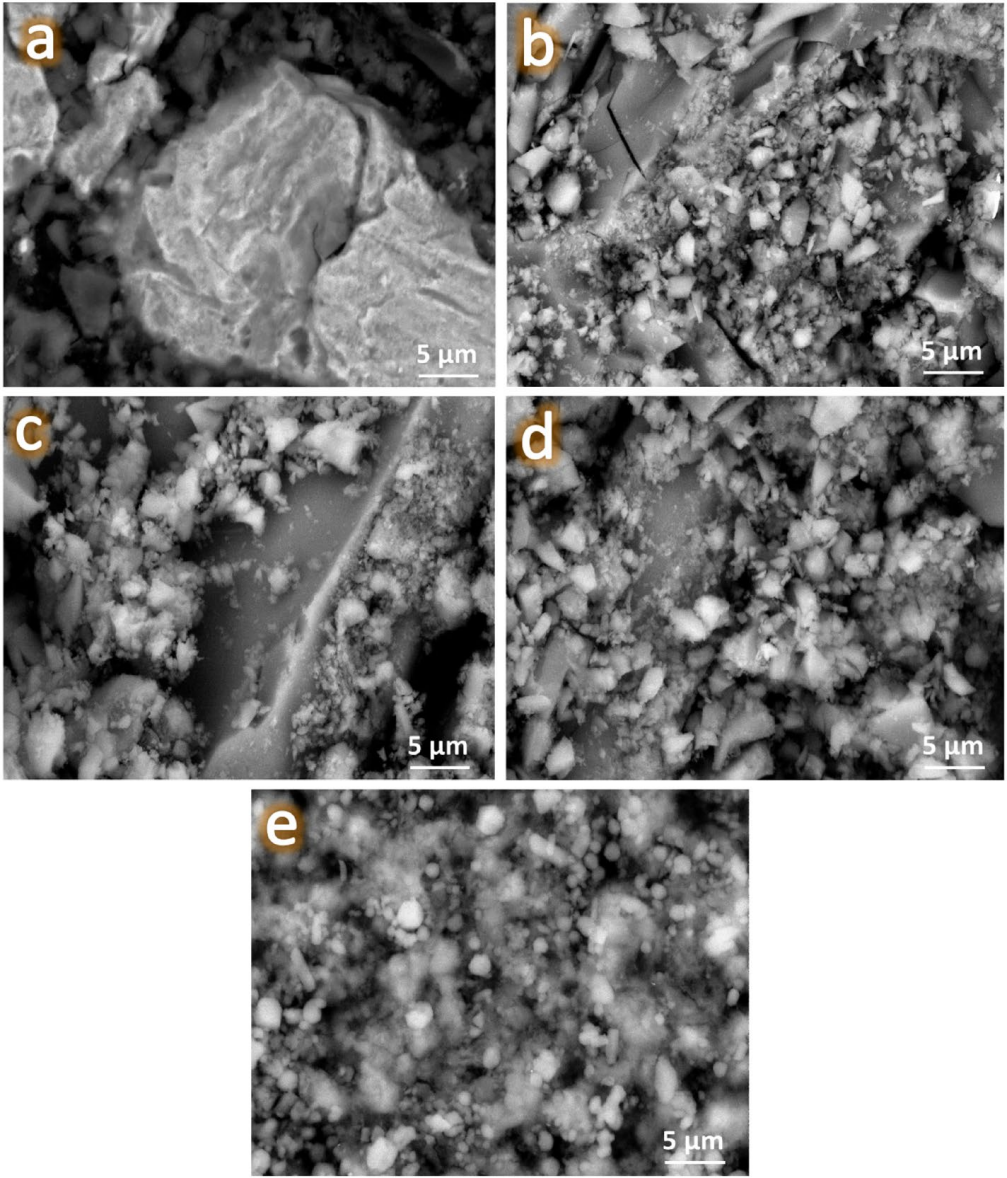
FESEM images of (**a**) ZM1, (**b**) ZM2, (**c**) ZM3, (**d**) ZM4, and (**e**) ZM5 samples after treatment in SBF solution for 14 days.

The capacity of a biomaterial to produce an HA layer (Ca_10_(PO_4_)_6_(OH)_2_) that resembles bone on its surface following immersion in an SBF solution is generally regarded as a test for determining a substance’s bioactivity. It is a straightforward and affordable procedure. It should be highlighted that the “bioactive property” refers to a material’s capacity to create the required layer. This suggests that the material, when transplanted into the human body, should exhibit good attachment to the surrounding live bone tissue^47^. This idea led to the 14-day incubation of all sintered samples in SBF solution. Then, as shown in Fig. 9a–e, they were submitted to FESEM to provide a reader with visual proof of the creation of the apatite layer on their surfaces.

By closely examining the image obtained in Fig. 9a, it can be noticed that the microstructure of the ZM1 sample is almost similar to that obtained before incubation in the SBF solution. This finding can be attributed to the absence of a bioactive phase, i.e., CaSiO_3_ used in this current study in the ZM1 sample despite the presence of MgZn_2_ and Mg_2_Zn_11_ phases which have a higher biodegradation behavior than pure Zn according to the literature^48^. In other words, the mentioned phases may improve the formation of the apatite layer on the surface of the sample due to its Mg content, but they cannot form the apatite layer alone in the absence of the biologically active phase. Regarding the ZM2 and ZM3 samples, a slight difference in bioactivity behavior was observed in favor of the ZM2 sample due to the presence of 1 vol%% Si_3_N_4_ in the ZM3 sample which resulted in a lower biodegradation rate of CaSiO_3_, MgZn_2_, and Mg_2_Zn_11_ phases as will be explained in the next section. By increasing the CaSiO_3_ phase content in ZM4 and ZM5 samples, the bioactive behavior also increased. Knowing the mechanism described in Ref.^49^ will help the reader to understand the relationship between increasing sample concentration of CaSiO_3_ and the development of an apatite condensate layer on its surface after soaking in SBF solution:

Calcium (Ca^2+^) ions are exchanged with hydrogen (H^+^) ions present in the solution when samples containing CaSiO_3_ are submerged in SBF, resulting in silanol (Si–OH) groups being formed on the surfaces of the samples. As a result, the presence of Si–O– groups on the surface results in the surface having a negative charge when the pH of SBF is raised close to the soaked sample. As a result of these subsequent actions, Ca^2+^ ions are easily drawn to this negatively charged surface and precipitate the desired layer, HA. The outstanding bioactive properties of CaSiO_3_ discovered are in good accord with those described in the literature^50,51^. In parallel to the positive effect of the CaSiO_3_ bioactive phase to develop the HA layer on the sample surface, Mg^2+^ ions are playing an assisting factor in encouraging this formation of the apatite layer according to the following equation:

9 $$ {\text{Mg}}_{{({\text{s}})}} + {\text{H}}_{{2}} {\text{O}}_{{({\text{aq}}.)}} \to {\text{Mg}}^{{{2} + }}_{{({\text{aq}}.)}} + {\text{2OH}}^{ - }_{{({\text{aq}}.)}} $$

This further enhances the production of the HA layer, which is important for bone-bonding ability, as a result of the dissolution of Mg^2+^ ions^52^. It should be highlighted that the HA layer resembles the mineralized stage of bone very much. As a result, there is a natural affinity between the collagen layer and the HA that aids in their fusion, allowing the collagen fibers in the bone to penetrate the activated HA layer and create a biological relationship^53^.

**Figure 10:**
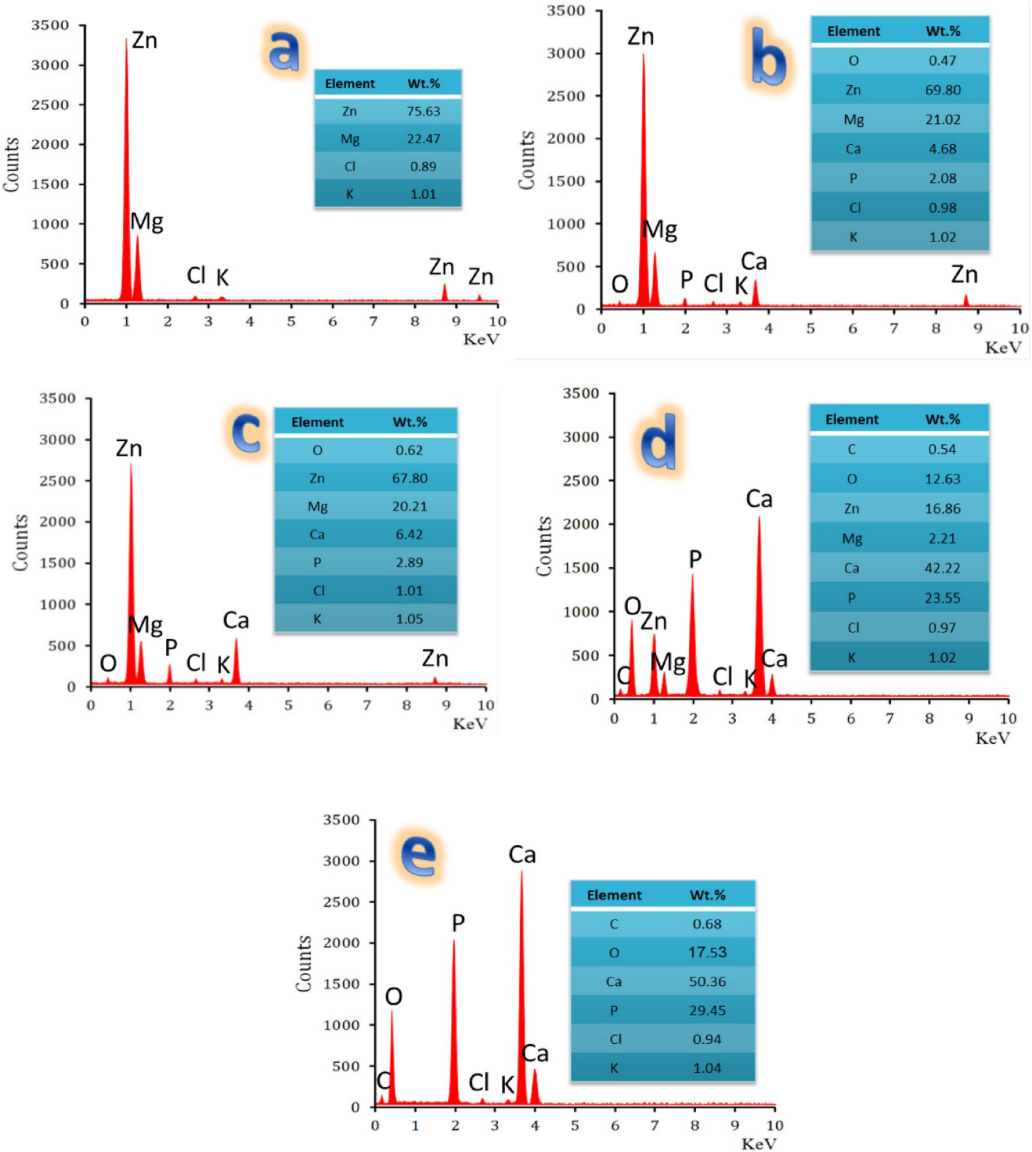
EDX spectra of the sintered samples, i.e., (**a**) ZM1, (**b**) ZM2, (**c**) ZM3, (**d**) ZM4, and (**e**) ZM5 after a 14-day treatment in SBF solution.

After 14 days in SBF solution, all treated samples underwent elemental analyses using the EDX technique, as seen in Fig. 10a–e. The following may be seen in EDX spectra:

- Sample ZM1’s EDX spectrum is mostly made up of Zn and Mg peaks. This shows that the sample does not have any biological activity.
- The height of successive Ca, P, and O peaks increases. It was shown that the ratio of Ca to P in the ZM5 sample approached 1.71, indicating the creation of a non-stoichiometric HA layer on its surface. It is important to note that for samples ZM2 and ZM3 samples, the ratio between Ca and P cannot be calculated accurately due to the insufficient complete coverage of the surface with the generated HA layer, which led to the calculation of Ca content present in the chemical composition of CaSiO_3_ present in the samples produced under the layer. This conclusion can be applied to sample ZM4 to a very small extent due to a very small proportion of the surface remaining uncovered by apatite, resulting in the Ca-to-P ratio increasing to 1.79, which is slightly higher than that for apatite.
- The apatite layer formed on the surface of the samples is carbonated hydroxyapatite (CHA; Ca_10_(PO_4_)_6_(CO_3_)_6_(OH)_2_) layer. This was inferred due to the presence of additional peaks; namely C and H.
- As the samples’ CaSiO_3_ content increased, a progressive drop in the height of the Si, Zn, Mg, and N peaks was seen as a result of the generated layer’s high coverage over the samples’ surface.
- Tiny peaks of Cl^−^ and K^+^ emerging from the SBF solution were noted.

#### Biodegradability tests

It is well-known that when an HA coating develops on sample surfaces, their masses fall. Therefore, weight loss measurement provides a helpful tool for monitoring the kinetics of conversion and sample dissolution^54^. Table 3 displays the percentages of weight loss for all tested samples after 14 days in the SBF solution.

**Table 3:** Percentage of weight loss for studied samples after immersion in SBF solution for 14 days.

Sample code	Weight loss (%)
ZM1	15.0
ZM2	18.1
ZM3	17.7
ZM4	22.3
ZM5	25.6

The results revealed that although the ZM1 specimen does not show any bioactive behavior, as can be seen in Fig. 9a, it loses about 15% of its weight. This result can be related to the results obtained from the XRD technique, which revealed the formation of MgZn_2_ and Mg_2_Zn_11_ phases which have a better decomposition rate compared to pure Zn. In light of these results, it can be argued that this weight loss occurring in the ZM1 sample could not be translated into the formation of the desired apatite layer but was due to the dissolution of these phases in the SBF solution. This weight loss increased in the ZM2 sample to about 18%, with a slight decrease in the ZM3 sample. In the remaining samples, namely ZM4 and ZM5, the weight loss continues to increase up to 25.6%. Because of the above, it can be concluded that the presence of Si_3_N_4_ plays a strong role in controlling the degradation of the prepared samples.

**Figure 11:**
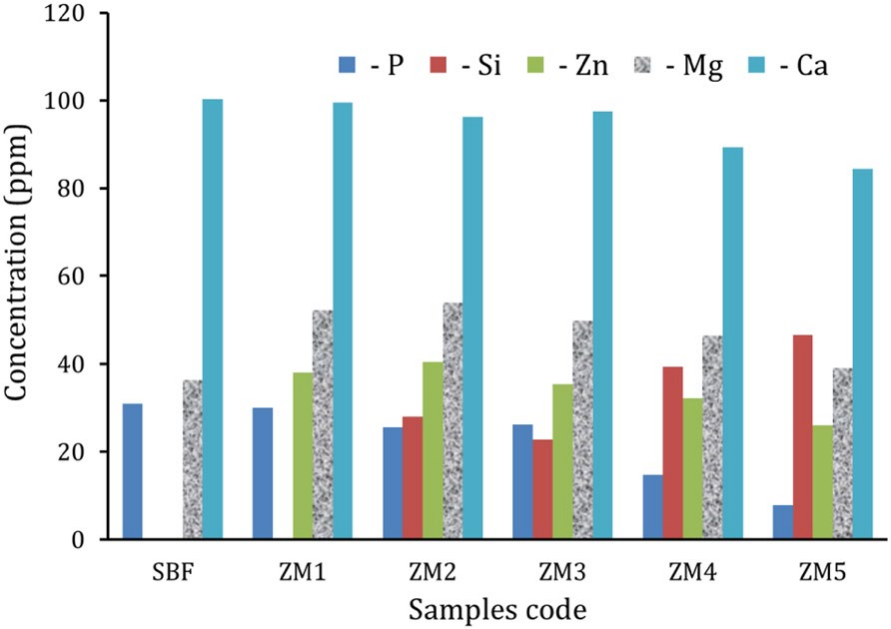
Variation in the concentrations of the P, Si, Zn, Mg, and Ca ions in the SBF solution after soaking of samples for 14 days.

ICP-AES was employed in this study for two key factors. To corroborate the findings about the bioactivity of the produced samples, it was first necessary to compare the concentrations of Ca^2+^ and P^3−^ ions before and after the samples had been soaked in SBF solution for 14 days. To check if the quantity of these ions released complies with global safety requirements, it was necessary to measure the release level of Si^4+^, Mg^2+^, and Zn^2+^ ions for all samples submerged in SBF solution for 14 days, as shown in Fig. 11 and listed in Table 4.

**Table 4:** Elemental concentrations in SBF solution before and after soaking all samples in SBF solution for 14 days.

Sample code	Elemental concentrations (ppm) ± 0.5				
	Zn	Mg	Si	Ca	P
SBF	–	36.4	–	100.2	30.9
ZM1	38.0	52.2	–	99.5	30.1
ZM2	40.5	53.9	28.1	96.3	25.5
ZM3	35.4	49.7	22.8	97.5	26.2
ZM4	32.2	46.5	39.4	89.3	14.8
ZM5	26.1	39.1	46.6	84.4	7.9

From Fig. 11, except for sample ZM1, it can be seen that the levels of Ca^2+^ and P^3−^ concentrations in the solution decrease continuously, which work with Ca^2+^ ions liberated from the samples to form an HA layer on the sample surface. In addition, the level of Si^4+^ ions appears in the ZM2 sample due to the dissolution of CaSiO_3_. Then, the level of Si^4+^ ions decreased slightly in the sample ZM3 due to the presence of 1 vol%% of Si_3_N_4_, which helps to increase the chemical durability of the prepared sample. The level of Si^4+^ ions increases due to the increase in the content of CaSiO_3_ and its solubility to form Si–OH groups, which contribute to the formation of an HA layer on the surfaces of the samples. On the other hand, the release of both Zn^2+^ and Mg^2+^ is negatively affected by the increase in Si_3_N_4_ content.

The findings demonstrated that almost all of the measured amounts of the aforementioned elements fall within the acceptable range. Because there are so few anecdotal reports of harm and a common assumption of safety, there is very little toxicity data available for aqueous Si^4+^ ingestion. However, a few research on rodents shows that dietary Si has a No Observed Adverse Effects Level (NOAEL) of 50,000 ppm (mg/l)^55^. Moreover, the typical daily dietary intake of Si is 20–50 mg for persons in Europe and North America. China and India have the lowest rates of hip fractures because they consume more Si (140–200 mg/day)^56^. On the other hand, Zn nanoparticles’ cytotoxic and genotoxic effects can be taken into account when their concentration equals 50 ppm^23^. Furthermore, the average adult has 24 g, or 1000 mmol, of Mg in their body, which equates to 20 mmol/kg of lean body mass. About 50–60% of Mg is found in muscles and other soft tissues. To keep extracellular Mg levels stable, around one-third of the Mg found in bones is accessible for exchange. The body’s extracellular Mg may be accounted for by less than 2% of Mg that is present in serum and red blood cells^57^. Despite these stated dose limitations of Zn and Mg elements, animal culture studies have shown that degradation of Zn–Mg alloys did not lead to any condition affecting the liver, heart, kidneys, or blood composition^35^.

#### Evaluation of cytotoxic activity of samples prepared using a human osteosarcoma cell line

**Figure 12:**
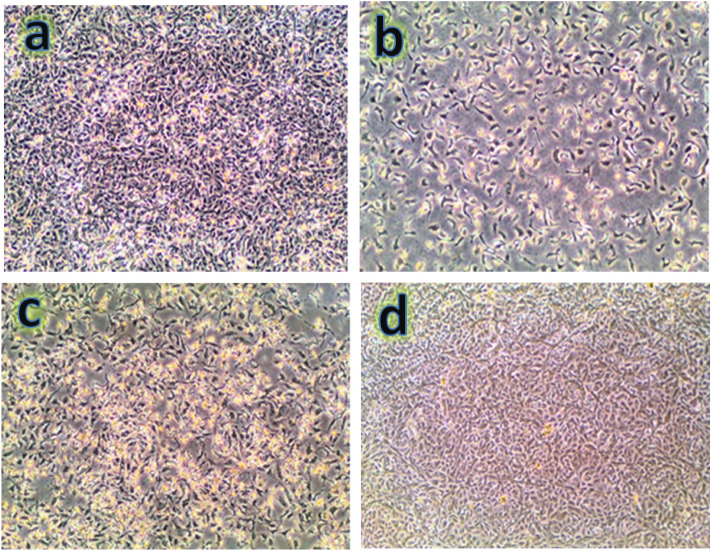
Images of osteosarcoma cells incubated for 2 day on (**a**) control, (**b**) ZM1, (**c**) ZM3, and (**d**) ZM5 nanocomposites.

The ability of a biomaterial to work, demonstrating a sufficient response without causing an allergic or inflammatory response during a specific application, is known as biocompatibility. A live creature or system (microenvironment) is referred to as a “host”, and the reaction of a host toward an alien biomaterial is known as the “host-response”^58^. Thus, osteosarcoma cells in contact with ZM1, ZM3, and ZM5 samples had their cell availability measured and expressed in comparison to the control group, which consists of cells without tested samples as shown in Fig. 12a–d. Table 5 displays the percentages of dead cells as a result of the action of the tested samples. The percentage of dead cells in contact with ZM1, ZM3, and ZM5 samples is 21.3, 16.5, and 9.2%, respectively. This means that the samples tested have good biocompatible behavior and are safe for use in bone replacement applications. It is important to note that this good biocompatibility is closely related to the results discussed in the previous section, i.e., the biodegradation test, as observed by the decrease in the percentage of dead cells with increasing ceramic additives, which serves to control the biodegradability of the samples tested and then increase their biocompatibility.

**Table 5:** Samples examined in comparison to human osteosarcoma cell lines.

Sample code	IC_50_ (µg/ml)	IC_90_ (µg/ml)	Remarks
Control	–	–	0%
ZM1	–	–	21.3% at 100 ppm
ZM3	–	–	16.5% at 100 ppm
ZM5	–	–	9.2% at 100 ppm

IC50: The sample’s lethal dose at which 50% of cells die within 48 h.

IC90: The sample’s lethal concentration, which results in 90% of cells dying in 48 h.

### Measurement of the different properties of the obtained samples

#### Physical properties

**Figure 13:**
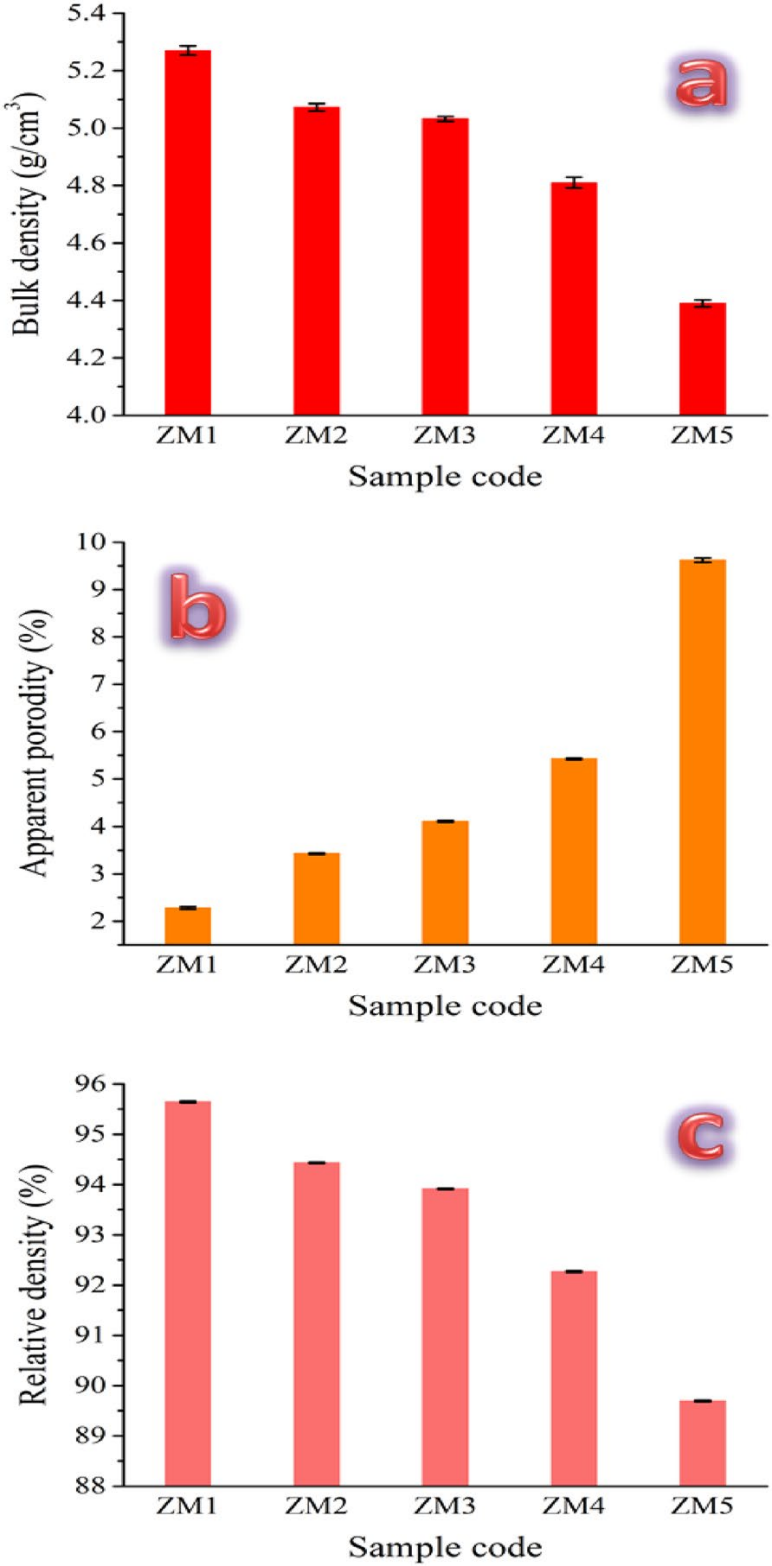
(**a**) Bulk density, (**b**) apparent porosity, and (**c**) relative density of all prepared samples after sintering at 360 °C for 1 h.

Figure 13a–c illustrates the bulk density, apparent porosity, and relative density of all samples, respectively. Furthermore, the mean value, standard deviation, and variance of the measured bulk density, relative density, and apparent porosity are listed in Table 6. The results show that the gradual rise in CaSiO_3_ and Si_3_N_4_ content plays a significant part in lowering the bulk and relative densities of the samples. This figure shows that by gradually increasing the amounts of CaSiO_3_ (from 0 to 20 vol%) and Si_3_N_4_ (0 to 4 vol%), the densities fell by 3.77, 4.71, 9.05, and 16.98%. It is important to note that this drop in bulk density can be explained by considering two factors. On the one hand, replacing a heavier material, i.e., Zn (7.13 g/cm^3^) with lighter ceramic materials, i.e., CaSiO_3_ (2.85 g/cm^3^) and Si_3_N_4_ (3.17 g/cm^3^). On the other hand, as discussed in “Surface morphology” section, there is a large difference in sintering temperature between ceramic materials and Zn–Mg alloy. To clarify this important point for the reader, the sintering mechanism must be presented, which can be summarized as follows:

**Table 6:** Statistical analyses of bulk density, relative density, and apparent porosity for all samples tested.

Sample	Mean value	Standard deviation	Variance
Bulk density (g/cm^3^)			
ZM1	5.27	0.01581	2.5E−4
ZM2	5.072	0.01304	1.7E−4
ZM3	5.032	0.00837	7E−5
ZM4	4.81	0.01871	3.5E−4
ZM5	4.39	0.01225	1.5E−4
Relative density (%)			
ZM1	95.644	0.01517	2.3E−4
ZM2	94.432	0.00837	7E−5
ZM3	93.914	0.0114	1.3E−4
ZM4	92.268	0.01483	2.2E−4
ZM5	89.694	0.01517	2.3E−4
Apparent porosity (%)			
ZM1	2.28	0.02646	7E−4
ZM2	3.43	0.01581	2.5E−4
ZM3	4.11	0.01581	2.5E−4
ZM4	5.42	0.01871	3.5E−4
ZM5	9.62	0.04637	0.00215

As was previously mentioned in Ref.^59^, successful densification depends on the sintering temperature that is selected while carrying out the sintering process in three phases. Powders’ ability to compress makes contact creation easier in the beginning. By forming “necks” between them, the particles are secondarily intimately connected. It is noteworthy that these necks develop when the sintering temperature approaches two–thirds of the melting point. The remaining porosity is sealed up and the particles are fully bound, rendering them invisible separately.

The values obtained for the apparent porosity of samples ZM1, ZM2, ZM3, ZM4, and ZM5 are 2.5, 3.6, 4.1, 5.5, and 7.6%, respectively. From these results, it can be seen that the porosity of all prepared samples converges to that of compact human bone, ranging from 5 to 13%. The porosity did not significantly drop in the final sample, despite the ceramic components having risen to about 25%. This is because, as was previously said in “Characterization of nanopowders and their particle size using HRTEM” section, the presence of these ceramic additives in the nanoscale range contributes to improving the behavior of densification, since the nanoscale additives exhibit superior densification compared to the microscale ones. It is crucial to remember that, as long as their mechanical properties are not unduly compromised, these materials must have enough porosity to support osseointegeration, cell proliferation, and angiogenesis in order to be used in orthopedic applications. These results are well-aligned with those covered in “Surface morphology” section.

#### Mechanical properties

**Figure 14:**
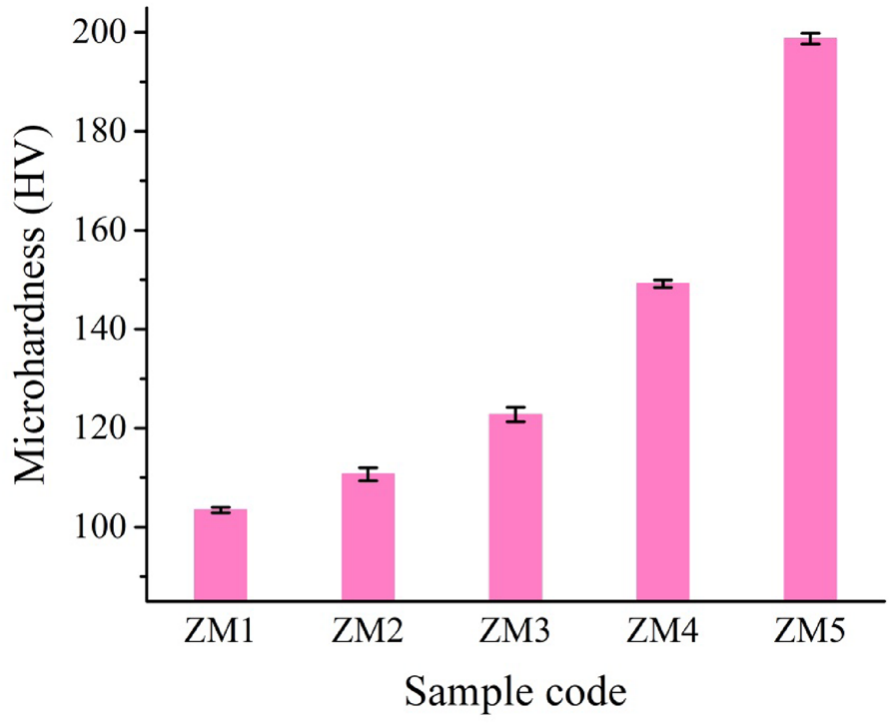
Microhardness of all samples prepared.

**Figure 15:**
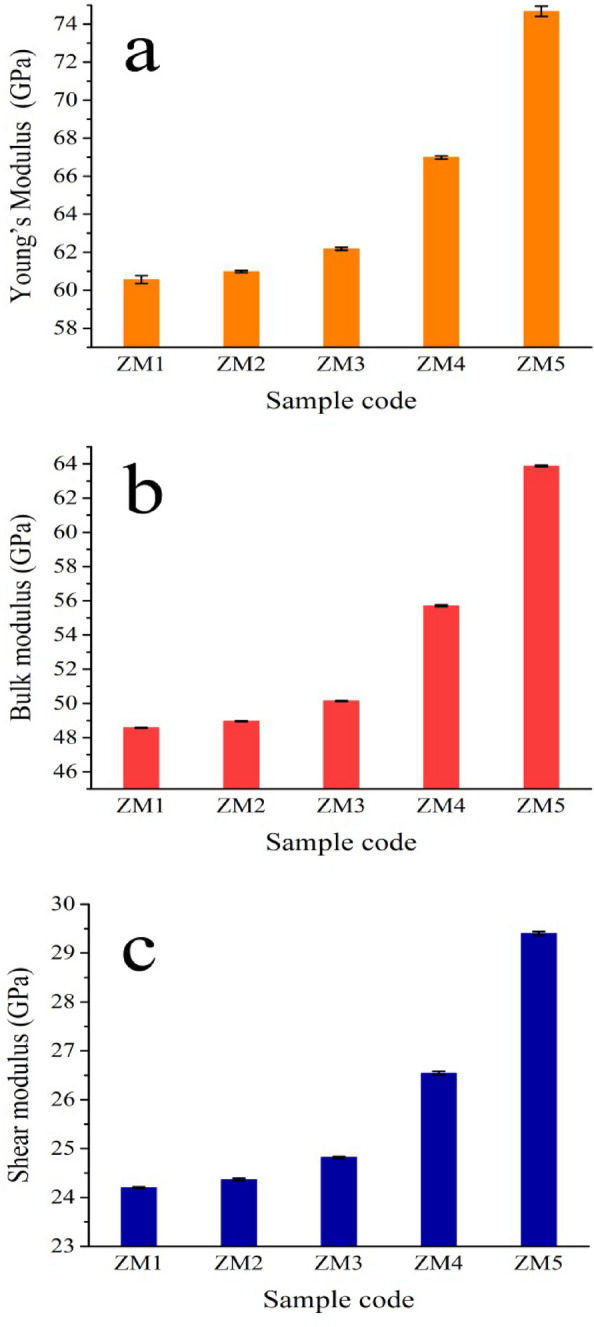
(**a**) Young’s modulus, (**b**) bulk modulus, and (**c**) shear modulus of all prepared samples.

Obtaining good mechanical properties that are strikingly comparable to those of human natural bone is generally considered to be one of the key objectives of creating nanocomposites for use in bone replacement applications. Because of its significance, the produced Zn–Mg alloy and its nanocomposites were measured in terms of microhardness, Young’s modulus, bulk modulus, and shear modulus as shown in Figs. 14 and 15a–c. In addition, the mean value, standard deviation, and variance of the measured mechanical properties are listed in Table 7. It is noteworthy to note that from these figures, the microhardness and other mechanical properties of the as-prepared nanocomposites follow an upward behavior as follows: ZM1 < ZM2 < ZM3 < ZM4 < ZM5. The microhardness values of samples ZM1, ZM2, ZM3, ZM4, and ZM5 are 105, 113, 125, 153, and 202 HV, respectively. This result is often because of several reasons. First, the successive increase in CaSiO_3_ and Si_3_N_4_ concentrations, which possess higher mechanical properties compared to the Zn–Mg alloy. Second, obtaining good densification as discussed in “Surface morphology” and “Physical properties” sections. Ultimately, as discussed in “Composition of phases” section, the formation of intermetallic phases, i.e., MgZn_2_ and Mg_2_Zn_11_ phases have better mechanical properties than pure Zn and Mg^16,33^.

**Table 7:** Statistical analyses of microhardness, Young’s modulus, bulk modulus, and shear modulus for all samples tested.

Sample	Mean value	Standard deviation	Variance
Microhardness (HV)			
ZM1	103.44	0.56	0.31
ZM2	110.7	1.33	1.76
ZM3	122.74	1.44	2.06
ZM4	149.21	0.74	0.55
ZM5	198.74	1.09	1.19
Young’s modulus (GPa)			
ZM1	60.56	0.20	0.0422
ZM2	60.976	0.06	0.00428
ZM3	62.18	0.08	0.00635
ZM4	66.98	0.09	0.0081
ZM5	74.672	0.27	0.07542
Bulk modulus (GPa)			
ZM1	48.57	0.026	6.7E−4
ZM2	48.96	0.027	7.2E−4
ZM3	50.14	0.031	8E−4
ZM4	55.70	0.051	0.00257
ZM5	63.88	0.044	0.0019
Shear modulus (GPa)			
ZM1	24.2	0.01732	3E−4
ZM2	24.366	0.02608	6.8E−4
ZM3	24.82	0.02121	4.5E−4
ZM4	26.544	0.03975	0.00158
ZM5	29.4	0.03937	0.00155

#### Tribology tests

**Figure 16:**
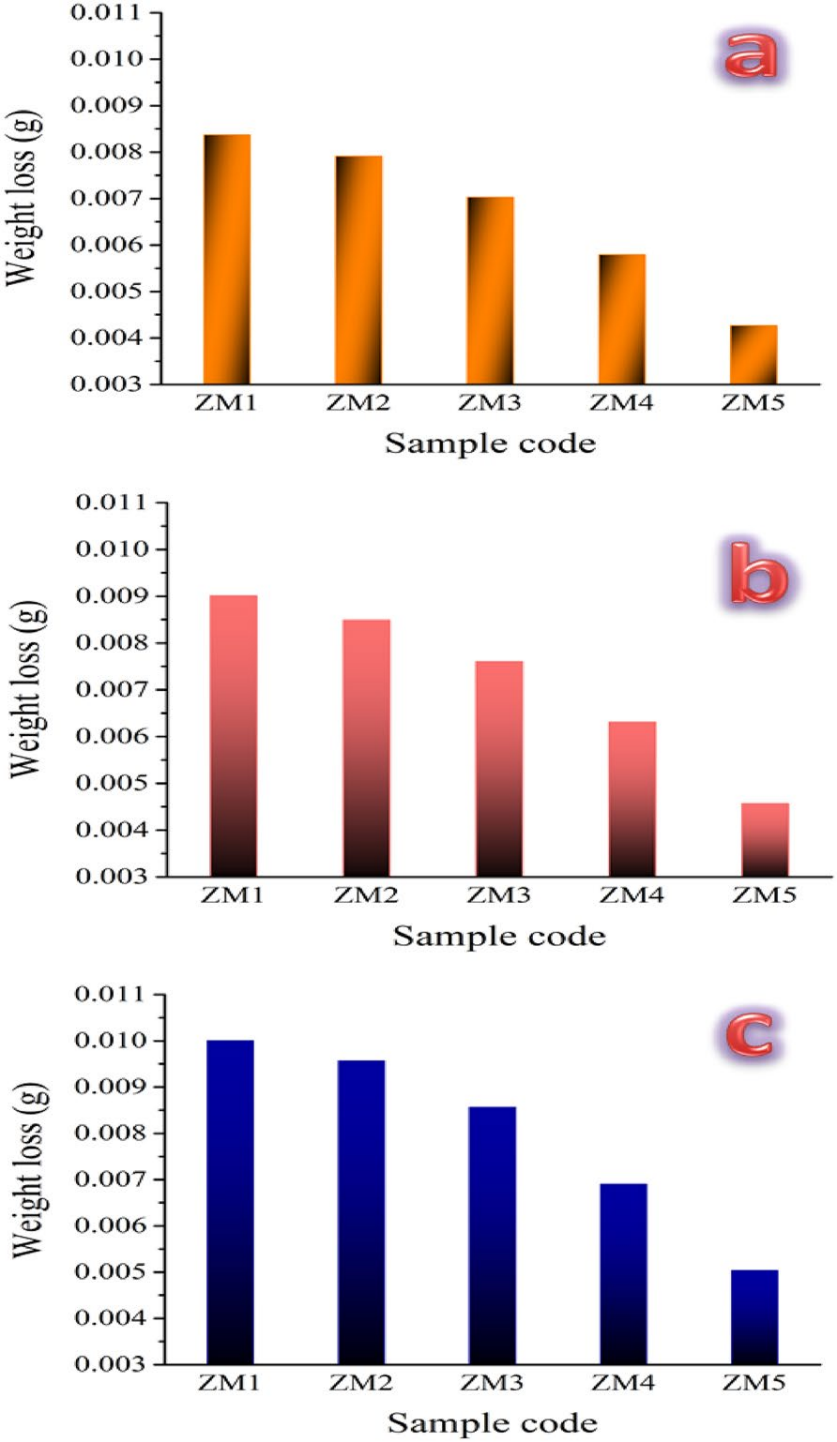
Weight loss for all sintered samples at three different loads, i.e., (**a**) 10 N, (**b**) 20 N, and (**c**) 40 N.

**Figure 17:**
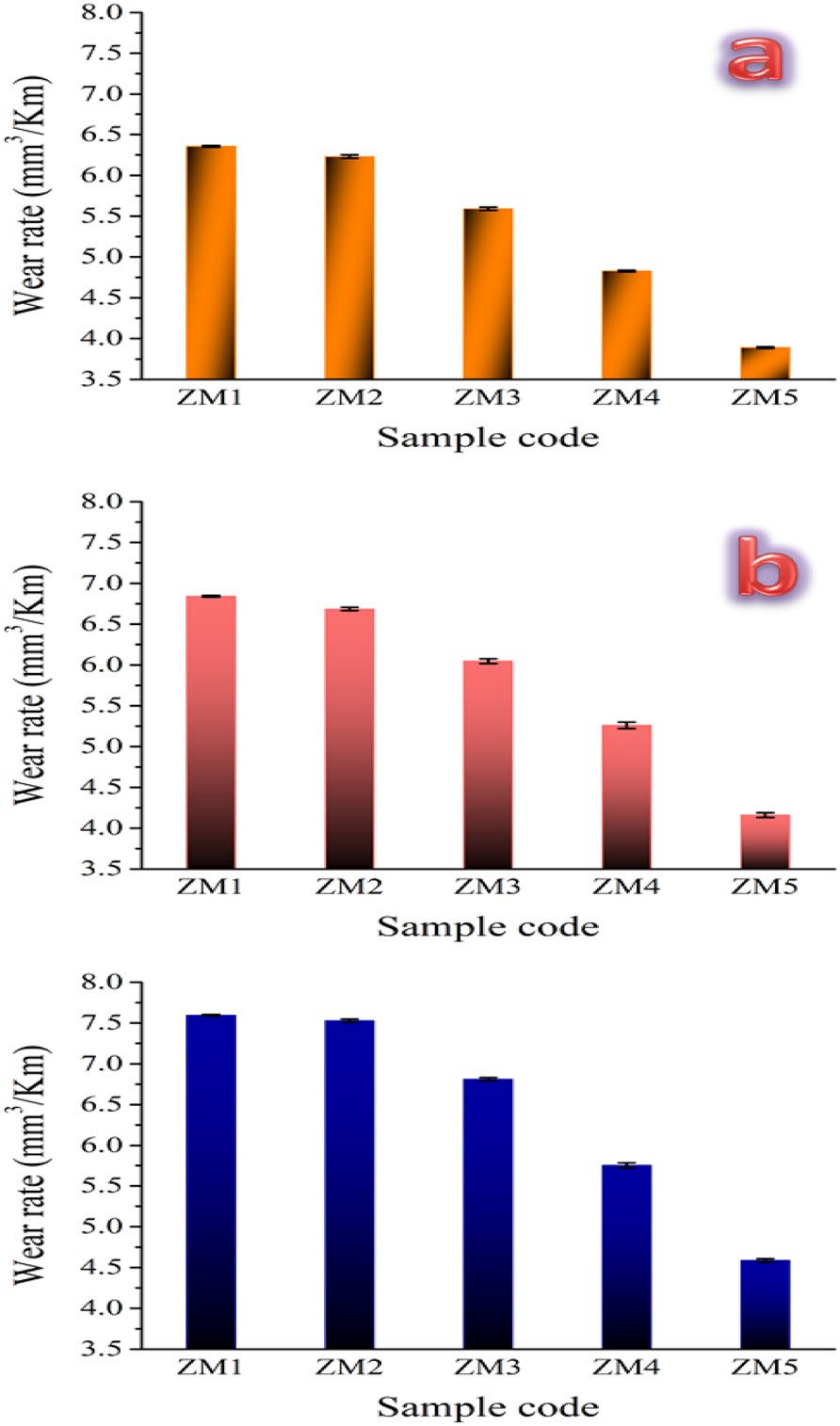
Wear rate for all sintered samples at three different loads, i.e., (**a**) 10 N, (**b**) 20 N, and (**c**) 40 N.

The highly reactive environment of the body causes metallic implants to corrode there. For instance, blood and other bodily fluids include many substances, such as proteins, salt, chlorine, and water, which make the body’s environment extremely reactive to metals^53^. Given that the biodegradable alloy that was produced, the biomaterial, degrades quickly and loses mechanical integrity, bringing further attention to this issue^60^. In this case, the weight loss and wear rate were assessed at various loads, namely 10, 20, and 30 N for all samples prepared and shown in Figs. 16a–c and 17a–c, respectively. Moreover, statistical analyses of the wear rate obtained at different applied loads are tabulated in Table 8. The obtained results reflected the role of ceramic additives, i.e., CaSiO_3_ and Si_3_N_4_, in enhancing the wear behavior of the prepared samples. This conclusion is evidenced by the results shown in Fig. 16, which display the improvement in weight loss for the prepared samples; namely ZM2, ZM3, ZM4, and ZM5, compared to the ZM1 sample by 7.05, 18.82, 40, and 62.35%, respectively when the applied load is 10 N. This improvement in weight loss continues even after raising the applied load to 20 N, as it is enhanced by 8.16, 19.38, 33.67, and 54.08% for the same samples mentioned above. In the same way, one may expect to obtain the same trend for the same samples after raising the applied load to 40 N, as they recorded improvements in weight loss by 4.54, 16.36, 29.09, and 49.09%. The same behavior was noticed for the recorded values of wear rate which is significantly improved by such an increase in the contents of CaSiO_3_ and Si_3_N_4_. Undoubtedly, this improvement in wear behavior can be attributed to several reasons. Specifically, the good densification, good mechanical properties, and the presence of two different types of ceramic materials are characterized by their good wear and corrosion resistance.

**Table 8:** Statistical analyses of wear rate for all prepared samples when different applied loads are applied, i.e., 10, 20, and 40 N.

Sample	Mean value	Standard deviation	Variance
Wear rate (10 N)			
ZM1	6.3556	0.01	1.268E−4
ZM2	6.23	0.02	2.5E−4
ZM3	5.59	0.02	6.5E−4
ZM4	4.828	0.01	3.2E−4
ZM5	3.888	0.01	1.7E−4
Wear rate (20 N)			
ZM1	6.842	0.01	1.7E−4
ZM2	6.686	0.02	3.3E−4
ZM3	6.046	0.03	0.00103
ZM4	5.26	0.04	0.0018
ZM5	4.16	0.03	7E−4
Wear rate (40 N)			
ZM1	7.596	0.01	1.3E−4
ZM2	7.53	0.02	4.5E−4
ZM3	6.812	0.02	8.2E−4
ZM4	5.752	0.03	6.7E−4
ZM5	4.586	0.02	4.3E−4

**Figure 18:**
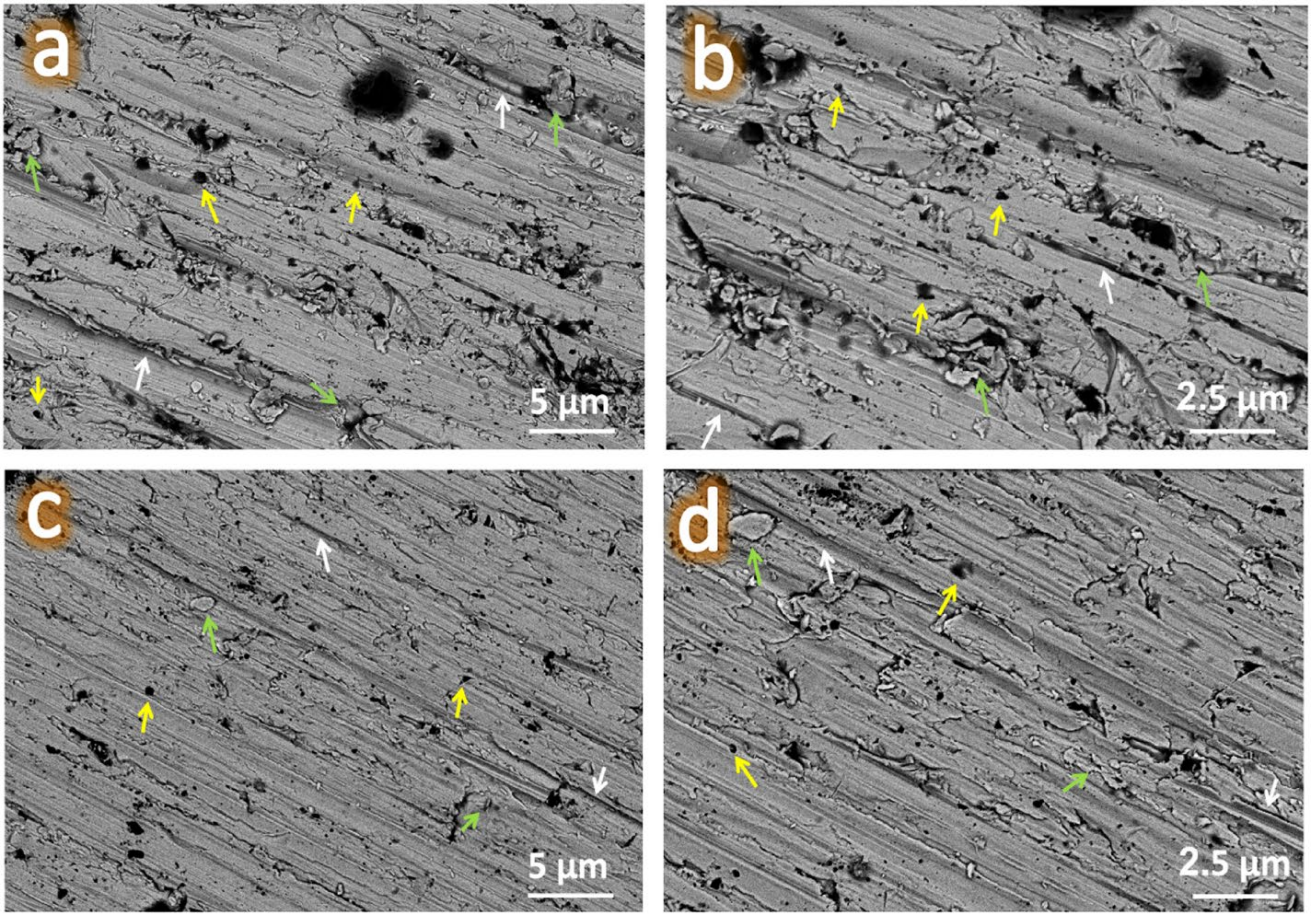
FESEM images of wear tracks under an applied load of 40 N for the ZM1 sample at (**a**) lower magnification and (**b**) higher magnification power, and the ZM5 sample at (**c**) lower magnification and (**d**) higher magnification power.

To assess the wear processes of Zn–Mg alloy and its nanocomposite, FESEM measurements were performed at two different magnifications on the worn surfaces of ZM1 and ZM5 samples at a sliding distance of 1500 m and under an applied load of 40 N, as shown in Fig. 18a–d. The only features on the wear track for the Zn–Mg matrix are loose layers and grooves, as seen in Fig. 18a, b. Surface delamination displays adhesive wear, including crack initiation, crack propagation, and materials’ ultimate breakage close to the surface. Figure 18c, d demonstrates that the surface of the ZM5 sample is smoother than that of the ZM1 sample, and the worn surface only exhibits occasional debris and minute grooves. Due to the poor microhardness of Zn–Mg alloy, some wear debris has flattened. In this wear track, very few fractures are visible; as a result, abrasive wear is the predominant wear mechanism^41^.

## Conclusions

Metallic materials are recognized to offer the right amount of strength, which opens the door for their use as implants in orthopedics and dentistry. The high rate of wear when placed in the human body and the poor ossointegration as a result of their failure to produce an HA layer on their surfaces are two very important issues that restrict the long-term effectiveness of these implants. In this context, a powder metallurgy approach was used to create a Zn-30% Mg intermetallic alloy. Then, the Zn–Mg alloy was thoroughly blended with CaSiO_3_ and Si_3_N_4_ in various volume percentages to enhance their biodegradability, bioactivity, and wear characteristics. The outcomes showed that the addition of CaSiO_3_ significantly improved the bioactivity behavior of the samples under study. Additionally, these materials’ improved biodegradability, wear resistance, and mechanical properties were achieved with the help of both CaSiO_3_ and Si_3_N_4_. These positive findings suggest that the produced materials can be successfully employed as implants in load-bearing bone applications.

## Data Availability

The datasets generated and/or analyzed during the current study are not publicly available because all data are presented in the article and therefore, there is no need to include raw data but they are available from the corresponding author upon reasonable request.
